# Dysfunctional breathing mimicking asthma exacerbation with acute hypoxemia: a case report

**DOI:** 10.1016/j.rmcr.2026.102419

**Published:** 2026-04-21

**Authors:** Kazuhiro Yamada, Kaho Hirai, Shiori Mizuoka, Kohei Mondori, Masashi Akioka, Kanako Sato, Hiroaki Nagamine, Yoshiya Matsumoto, Toshiyuki Nakai, Tetsuya Watanabe, Kazuhisa Asai, Tomoya Kawaguchi

**Affiliations:** a Department of Respiratory Medicine, Graduate School of Medicine, Osaka Metropolitan University, 1-4-3, Asahimachi, Abeno-Ku, Osaka, 545-8585, Japan; b Department of Respiratory Medicine, Osaka City General Hospital, 2-13-22 Miyakojima-hondori, Miyakojima-ku, Osaka, 534-0021, Japan

**Keywords:** Dysfunctional breathing, Nijmegen questionnaire, Brompton breathing pattern assessment tool, Asthma, Inducible laryngeal obstruction

## Abstract

**Background:**

Dysfunctional breathing (DB) is characterized by abnormal breathing patterns and is often under-recognized in clinical practice. Although DB is generally regarded to be a functional breathing disorder, its potential to cause clinically significant hypoxemia has not been well documented.

**Case report:**

A case involving a 24-year-old woman diagnosed with asthma and other psychiatric comorbidities, who developed acute wheezing and oxygen desaturation during hospitalization, is reported. Despite treatment with systemic corticosteroids, the patient's symptoms did not improve. Careful physical examination revealed wheezing, predominantly over the laryngeal area, and detailed observation of her breathing pattern demonstrated irregular respiration with intermittent breath-holding and alternating hyperventilation. The Nijmegen Questionnaire (NQ) score was below the conventional cut-off for DB, whereas the Breathing Pattern Assessment Tool (BPAT) score indicated DB. By adhering to simple bedside breathing instructions, her oxygen saturation rapidly improved without additional bronchodilator or corticosteroid therapy, and both wheezing and desaturation resolved.

**Conclusion:**

This case illustrates that DB can be associated with acute hypoxemia and may mimic asthma exacerbation. Careful observation of breathing patterns and the complementary use of the BPAT and NQ are valuable for accurate diagnosis. Simple breathing instructions may serve as an effective initial intervention, helping to avoid misdiagnosis and unnecessary corticosteroid treatment.

## Highlights

• Dysfunctional breathing is often misdiagnosed as asthma exacerbation, leading to overtreatment.
• Dysfunctional breathing can present with acute hypoxemia.
• Combined use of BPAT and NQ improves clinical recognition of dysfunctional breathing.

## 1 Introduction

Dysfunctional breathing (DB) is characterized by abnormal breathing patterns that occur either in the absence of organic diseases or secondary to cardiopulmonary or neurological conditions. Importantly, DB is not merely a psychiatric disorder but also a breathing-pattern abnormality that may occur with or without mental health comorbidities [1,2]. Despite its clinical relevance, DB is not always adequately recognized in routine clinical practice and, is therefore, frequently underdiagnosed or misdiagnosed, particularly in asthma [3,4]. The prevalence of DB in the general population in Japan has been reported to be 11.0%, indicating that it is not an uncommon condition [5]. To date, however, no gold-standard diagnostic criteria for DB have been established. The Nijmegen Questionnaire (NQ), originally developed to screen for hyperventilation syndrome, has been widely used for DB screening, with a score ≥23 commonly applied as the diagnostic threshold [6,7]. Notably, the validity of the NQ was recently confirmed in a Japanese population [8]. Although the NQ is the most commonly used screening tool for DB, it relies primarily on patient-reported symptoms.

In contrast, the Brompton Breathing Pattern Assessment Tool (BPAT) is a clinician-administered observational tool based on a 1 min assessment of breathing at rest. It consists of 7 items, with a total score ranging from 0 to 14, and higher scores indicating a more abnormal breathing pattern. A BPAT score ≥4 has been suggested to indicate a breathing pattern disorder or DB [9].

Awareness of DB remains limited in current clinical practice. Among patients with asthma, DB has been associated with impaired quality of life and an increased risk for overtreatment due to under-recognition [7]. Given the high prevalence of DB among patients with asthma (31%) [5], overtreatment may be more common. Although DB is generally considered to be a functional breathing disorder, its potential to cause clinically significant hypoxemia has not been well documented.

Herein, we report a case involving a patient diagnosed with asthma in whom DB was associated with acute hypoxemia. This case highlights the importance of careful observation of breathing patterns and the use of the BPAT and NQ for identifying DB.

## 2 Case report

A 24-year-old woman was admitted to the psychiatric ward of the authors’ hospital with poor oral intake associated with psychological stress. Her medical history included dissociation, autism spectrum disorder, and attention-deficit/hyperactivity disorders. She was diagnosed with asthma and received inhaled fluticasone/umeclidinium/vilanterol once daily, as well as montelukast, tezepelumab, and fexofenadine. Additionally, she was taking multiple psychotropic medications, including alprazolam, lamotrigine, trazodone, rilmazafone, duloxetine, aripiprazole, and biperiden. She had no history of smoking or alcohol consumption, and had been caring for 2 indoor dogs for 10 years, with no relevant family history.

On day 2 of hospitalization, she repeatedly inhaled procaterol 55 times over a short period due to dyspnea. She subsequently developed palpitations and wheezing, prompting a request for consultation with the respiratory medicine department. On initial examination, the patient was alert and her vital signs were as follows: body temperature, 36.6 °C; blood pressure, 94/64 mmHg; heart rate, 124 beats/min; respiratory rate, 20 breaths/min; and oxygen saturation (SpO_2_), 91% on room air. Physical examination revealed wheezing predominantly over the neck and upper airway rather than the lung fields. Cardiac examination results were unremarkable. No peripheral edema or jugular venous distension was observed.

**Fig. 1:**
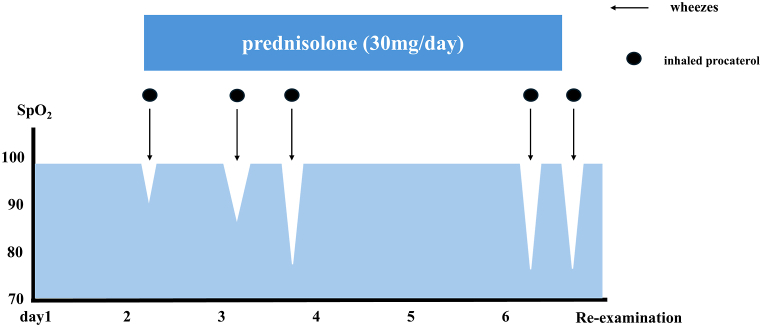
Clinical course. Clinical course illustrating intermittent oxygen desaturation despite systemic corticosteroid therapy. Wheezing and transient decline in oxygen saturation (SpO_2_) were observed during hospitalization, with no sustained response to prednisolone or inhaled bronchodilators.

**Fig. 2:**
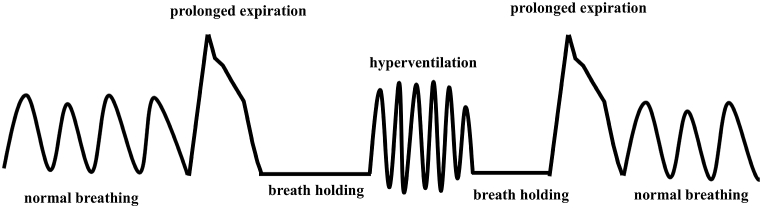
Schematic illustration of abnormal breathing patterns observed in this case. Schematic illustration of abnormal breathing patterns observed in this case, including intermittent breath holding, prolonged expiration, and episodes of hyperventilation, consistent with dysfunctional breathing.

Her white blood cell count was mildly increased (10,900 cells/μL), whereas inflammatory marker levels were within the normal ranges (C-reactive protein, 0.05 mg/dL). Serum electrolyte analysis revealed hypokalemia (potassium, 2.9 mEq/L). Arterial blood gas analysis on 1 L/min oxygen via nasal cannula revealed mild acidemia (pH 7.337), an elevated partial pressure of oxygen (128 Torr), a normal partial pressure of carbon dioxide (39.1 Torr), reduced bicarbonate (13 mEq/L), and an elevated lactate level (4.2 mmol/L), consistent with metabolic acidosis with lactate accumulation. One month before admission, her eosinophil count was 0%, total serum immunoglobulin E level was 4.6 IU/mL, and fractional exhaled nitric oxide was 13 parts per billion. Chest radiography revealed no abnormalities. Despite treatment with prednisolone (30 mg/day), her clinical condition did not improve and she developed recurrent episodes of sudden wheezing accompanied by oxygen desaturation. Therefore, further reassessment was performed on hospital day 6 (Fig. 1), during which her oxygen saturation (SpO_2_) transiently decreased to 83%. Wheezing was most prominent in the laryngeal area. Careful observation of her breathing pattern revealed irregular respiration characterized by alternating breath holding and hyperventilation, with an inspiratory-to-expiratory ratio of approximately 1:1. Expiration was prolonged only during periods when wheezing was auscultated (Fig. 2). These findings were inconsistent with typical asthma exacerbations and raised suspicion for a functional breathing disorder. To further evaluate this possibility, the NQ and BPAT were administered. The NQ score was 18, which did not meet the conventional cut-off for DB, whereas the BPAT score was 7, which did fulfill the criteria for DB. After informing the patient that her breathing pattern was irregular, guided breathing instructions encouraging gentle inspiration and slow expiration were provided at the bedside. Her oxygen saturation rapidly improved from 83% to 98% within approximately 1 min, without additional steroid or bronchodilator therapy. Based on overall clinical presentation, breathing pattern characteristics, questionnaire assessments, and rapid response to breathing instructions, DB was diagnosed instead of asthma exacerbation. From that day onward, wheezing and oxygen desaturation resolved without the further use of prednisolone.

## 3 Discussion

Two important clinical issues were identified in the present case. This case underscores that DB may present with acute hypoxemia and highlights the importance of combining objective evaluations using the BPAT with subjective assessments using the NQ.

First, DB is generally regarded to be a functional breathing disorder and is associated with clinically significant hypoxemia. DB is often assumed not to cause oxygen desaturation; however, this assumption may lead to the under-recognition of clinically relevant hypoxemia in patients with DB. DB encompasses a variety of abnormal breathing patterns, including shallow breathing, prolonged expiration, breath-holding, and irregular respiratory rhythm, which may coexist in a single patient [2]. In the present case, irregular breathing characterized by intermittent breath-holding was directly observed, suggesting that abnormal breathing patterns in DB could possibly impair effective ventilation and lead to hypoxemia. In addition, psychotropic medications, particularly sedatives, such as benzodiazepines, may blunt ventilatory responses to hypoxia and contribute to hypoxemia [10]. Moreover, psychiatric conditions may alter the perception of dyspnea, potentially delaying the recognition of clinically significant oxygen desaturation [11]. Collectively, these findings suggest that DB contributed to hypoxemia in the present case, which was potentially influenced by the underlying psychiatric background.

As an alternative explanation for desaturation, wheezing localized to the laryngeal area raises the possibility of inducible laryngeal obstruction (ILO), also known as vocal cord dysfunction, and is frequently associated with DB and has been reported to cause hypoxemia during acute episodes [12]. In the present case, the expiratory wheezing appeared to be inconsistent with ILO, which is classically associated with inspiratory stridor. However, expiratory-only presentations of ILO have been acknowledged, albeit less well studied [[13], [14], [15]]. Wheezing localized in the laryngeal area raises the possibility of ILO, which may contribute to hypoxemia in the setting of an abnormal breathing pattern. However, in routine clinical practice, direct assessment of the laryngeal area is often difficult, and the presence of ILO cannot be definitively confirmed. DB is a heterogeneous condition that includes ILO and abnormal breathing patterns. Importantly, abnormal breathing patterns characteristic of DB may contribute to hypoxemia, regardless of the presence of ILO. Accordingly, clinicians should recognize DB as a potential cause of hypoxemia to avoid misdiagnosis and to facilitate appropriate management, especially in asthma patients who show a DB of 31% [5]. Second, combining objective evaluation using the BPAT with subjective assessment using the NQ is important for identifying DB. The BPAT is an objective assessment tool comprising 7 items related to the following abnormal breathing patterns: irregular respiratory rhythm; tachypnea at rest; predominant upper chest breathing; use of accessory respiratory muscles; shallow breathing; frequent sighing or deep breaths; and mouth breathing during quiet respiration [9]. In the patient described, the BPAT score was 7, indicating objectively abnormal breathing patterns. Careful observation of the patient's breathing pattern enabled us to identify DB using the BPAT and exclude asthma exacerbation.

The NQ is a subjective evaluation that has been widely used for the screening and diagnosis of DB in research settings. The NQ consists of the following 16 items: chest tingling/pain; body stiffness or tightness; blurred vision; dizziness; inability to concentrate; deep breathing; shortness of breath; chest tightness; bloated (upset) stomach; tingling fingers; inability to breathe deeply; stiffness in the fingers; tightness around the mouth; cold hands or feet; palpitations; and insomnia. These items reflect symptoms across 4 domains—namely, respiratory, anxiety, peripheral, and central neurovascular systems—with each item assessed on a five-point scale (0–4, total score 0–64) [16].

In the present case, the NQ score was 18, below the cut-off (≥23) for DB. This subthreshold score may reflect the underreporting of anxiety-related symptoms on self-report questionnaires due to the stigma associated with mental health problems or socially desirable responding [17,18]. Therefore, cautious interpretation of NQ results may be required in patients with psychiatric conditions. Consistent with previous asthma research reporting that the BPAT and the NQ are insufficient when used alone to identify DB [13], this case suggests that their combined use is important and may be particularly essential for patients with psychiatric comorbidities.

Wheezing with concomitant oxygen desaturation is easily mistaken for asthma exacerbation, and the failure to identify DB may result in prolonged corticosteroid therapy. Therefore, timely recognition and appropriate treatment of DB are crucial. DB is typically treated using multidisciplinary programs that incorporate breathing retraining, patient education, and relaxation techniques. The Papworth method is an example of such an approach, combining diaphragmatic breathing with slow nasal expiration, education regarding stress–breathing interactions, relaxation training, and integration into daily activities supported by home practice [19]. However, these approaches are often difficult to implement in clinical settings. In this case, simple instructions to restore normal breathing patterns (breathing control or retraining) were effective. As an initial approach, breathing instructions may be the most practical strategy when dysfunctional breathing is suspected. This approach may facilitate an accurate diagnosis while helping to rule-out asthma exacerbations.

## 4 Conclusions

DB is associated with clinically significant hypoxemia. Careful observation of breathing patterns, including assessment using the BPAT, is crucial to distinguish DB from asthma exacerbation and avoid the unnecessary use of systemic corticosteroids such as prednisolone. Increasing awareness of DB among medical professionals is essential to prevent misdiagnosis and ensure appropriate management. Incorporating breathing pattern assessment may improve diagnostic accuracy in patients presenting with wheezing and hypoxemia.

## CRediT authorship contribution statement

**Kazuhiro Yamada:** Conceptualization, Data curation, Writing – original draft, Writing – review & editing. **Kaho Hirai:** Conceptualization, Writing – review & editing. **Shiori Mizuoka:** Writing – review & editing. **Kohei Mondori:** Writing – review & editing. **Masashi Akioka:** Writing – review & editing. **Kanako Sato:** Writing – review & editing. **Hiroaki Nagamine:** Writing – review & editing. **Yoshiya Matsumoto:** Writing – review & editing. **Toshiyuki Nakai:** Writing – review & editing. **Tetsuya Watanabe:** Writing – review & editing, Supervision. **Kazuhisa Asai:** Writing – review & editing, Supervision. **Tomoya Kawaguchi:** Writing – review & editing.

## Consent for publication

Written informed consent was obtained from the patient for publication of this case report.

## Declaration of competing interest

The authors declare that they have no known competing financial interests or personal relationships that could have appeared to influence the work reported in this paper.
